# Supplementary material for: Molecular and morphological congruence of three new cryptic Neopetrosia spp. in the Caribbean
Source: PeerJ. 2019 Feb 5;7:e6371. doi: 10.7717/peerj.6371 (PMC6368163; doi:10.7717/peerj.6371)
Supplement: Supplemental Information 6 [file peerj-07-6371-s006.docx]

| **Gene** | **Primer name** | **5'---------->3' sequence** | **References** |
| --- | --- | --- | --- |
| COI | LCO1490 | GGTCAACAAATCATAAAGATATTGG | Folmer *et al.* 1994 |
|  | HCO2198 | TAAACTTCAGGGTGACCAAAAAATCA | Folmer *et al.* 1994 |
|  | COINPIIIRV | AGTAGTATTAAAGTTTCTGTCGG | This study |
|  | COINPIIISPECF1 | GACCCTCTATTTATTGTTTGGAG | This study |
|  | COINPIIRV1 | ATGTTGGTACTATTTGCGATACGA | This study |
|  |  |  |  |
| 28S rRNA | LSU5F | TAGGTCGACCCGCTGCCYTTAAGC | Olsen (NHM London, Pers. Comm) |
|  | LSU300RV | CAACTTTCCCTCACGGTACTT | Olsen (NHM London, Pers. Comm) |
|  | C2-fwd | GAAAAGAACTTTGRARAGAGAGT | Chombard *et al.* 1998 |
|  | D2-rev | TCC GTG TTT CAA GAC GGG | Chombard et al. 1998 |
|  | NPRV2 | ACCCAAGTGCGACGATCGATTT | This study |
|  | NPFWD2 | ACCGATAGCGAACAAGTACCG | This study |
|  |  |  |  |
| 18S rRNA | HomoR | CTTGTTACGACTTTTACYTCCTC | Kelly-Borges & Pomponi 1994 |
|  | F | CTGGTGCCAGCAGCCGCGG | Kelly-Borges & Pomponi 1994 |
|  | NPIIRV4 | TTATTGCCTCAGTCCTCCCCC | This study |
