# Supplementary figures and images for: Molecular and morphological congruence of three new cryptic Neopetrosia spp. in the Caribbean
Source: PeerJ. 2019 Feb 5;7:e6371. doi: 10.7717/peerj.6371 (PMC6368163; doi:10.7717/peerj.6371)

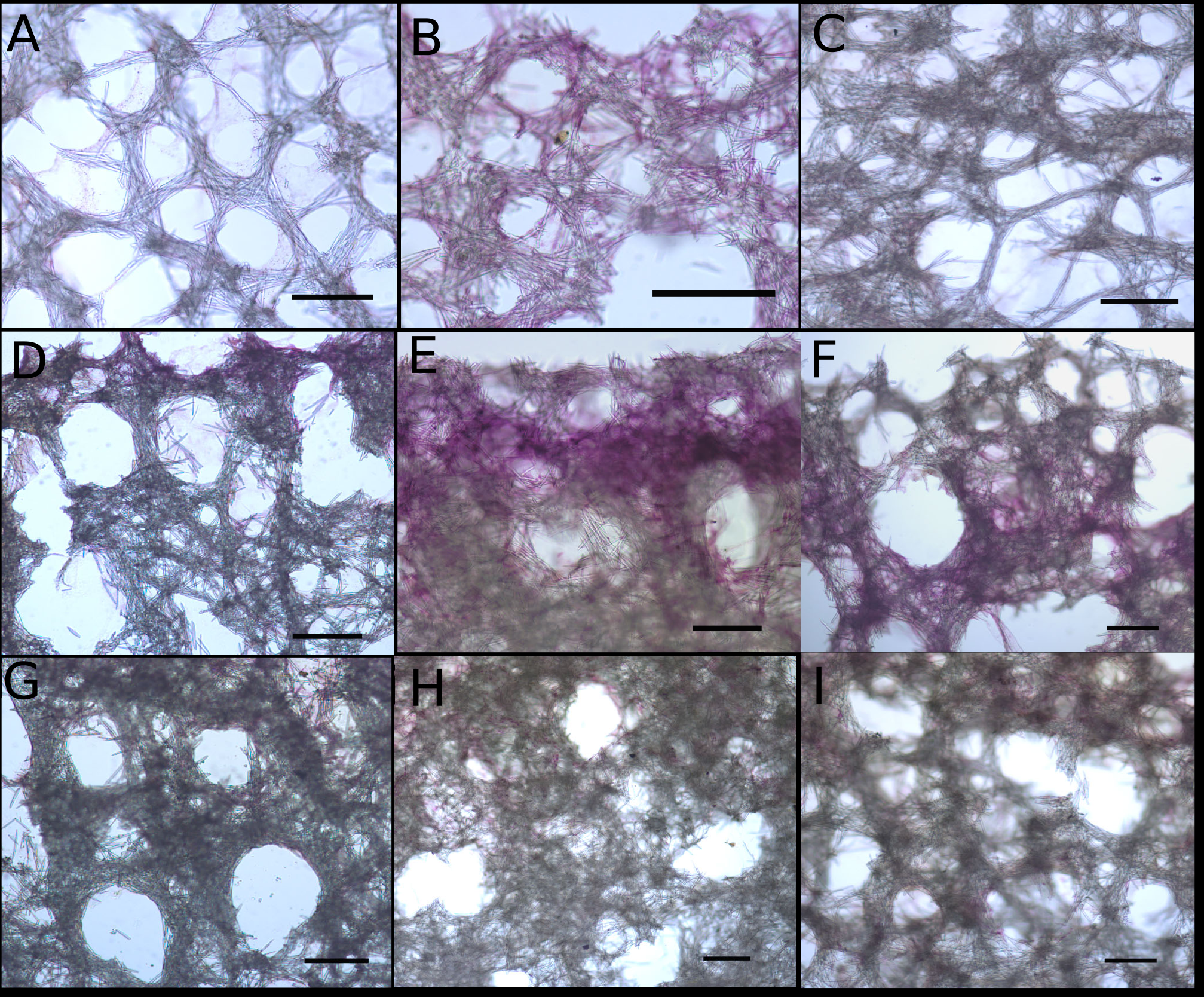

Supplement: Supplemental Information 9 — Close-up images of tangential sections of Panama specimens (A) UF 3856 (B) UF 3858, and (C) UF 3860, with corresponding (from second and third rows) images of (D–F) perpendicular sections through the ectosome and choanosome (LM), and close-up image of the choanosome (G–I) (LM). Scale bar in all images is 300 μm. [file peerj-07-6371-s009.png]

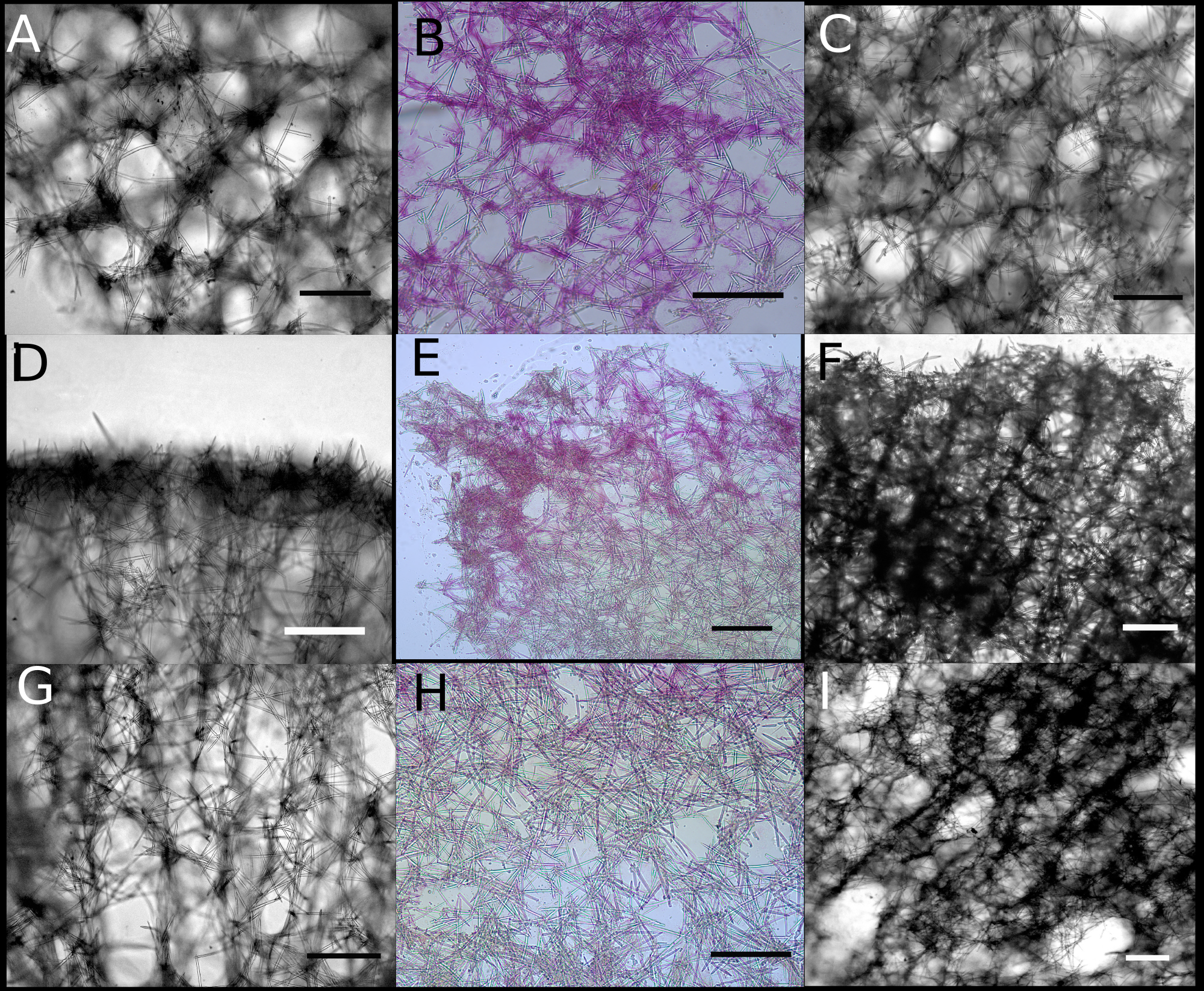

Supplement: Supplemental Information 10 — Close-up images of tangential sections from individual PPA07 (A) and the holotype UF 3854 (B), and of the paratype ICN-MHN(Po) 0269 (C) from Santa Marta, Colombia, with corresponding (from second and third rows) images of (D–F) perpendicular sections through the ectosome and choanosome (LM), and close-up image of the choanosome (G–I) (LM). Scale bar in all images is 200 μm. [file peerj-07-6371-s010.png]

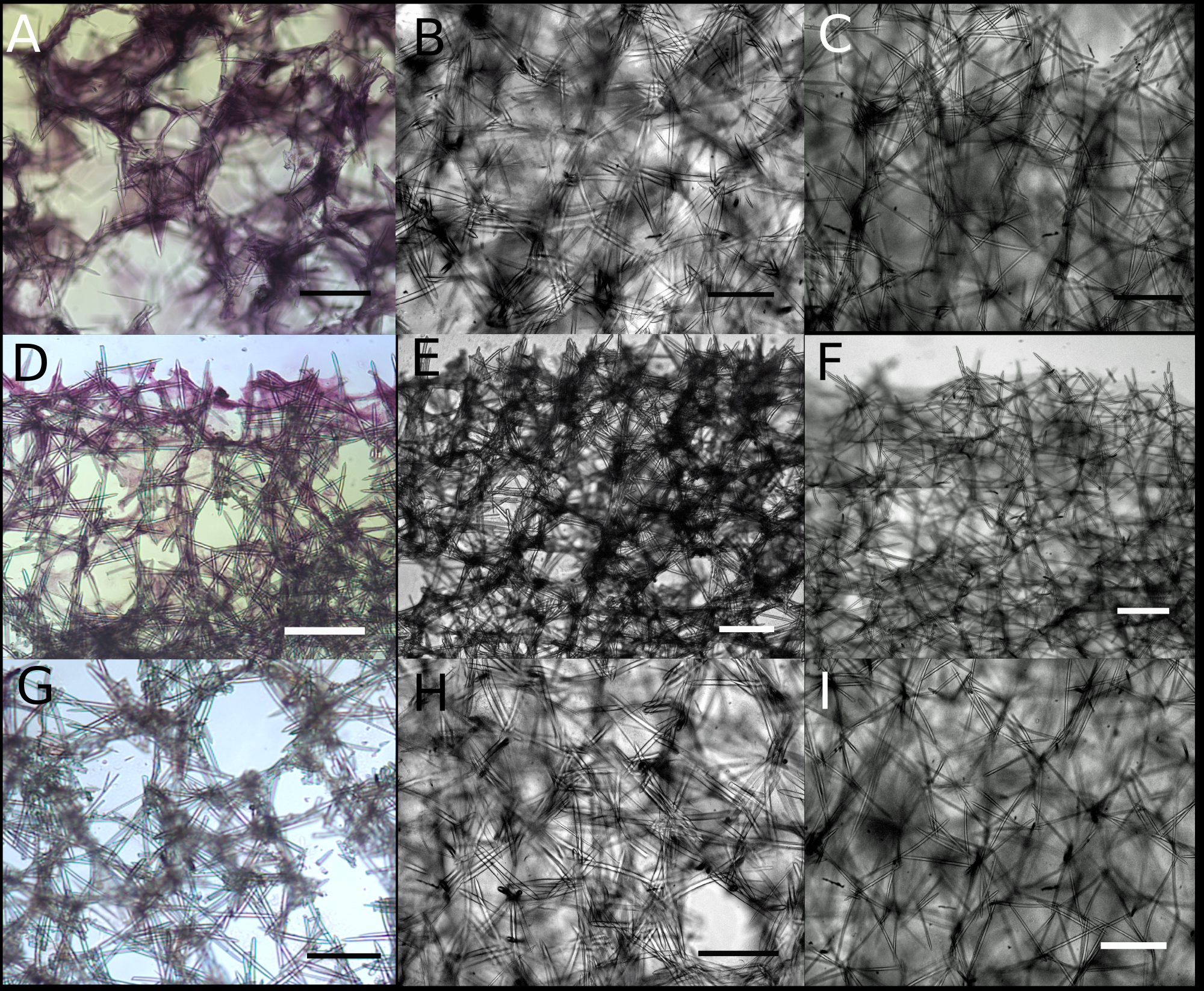

Supplement: Supplemental Information 11 — Close-up images of tangential sections from the holotype UF 3857 (A), paratype PPA 38 (B), and paratype SZ-21 from Martinique (C), with corresponding (from second and third rows) images of (D–F) perpendicular sections through the ectosome and choanosome (LM), and close-up image of the choanosome (G–I) (LM). Scale bar in all images is 200 μm. [file peerj-07-6371-s011.png]
